# Retrospective study assessing outcomes in cardiac surgery after implementation of Quantra

**DOI:** 10.1186/s13019-023-02245-x

**Published:** 2023-04-17

**Authors:** Pierre Tibi, Jess Thompson, Saina Attaran, Elizabeth Black

**Affiliations:** Department of Cardiovascular Surgery, Yavapai Regional Medical Center, 811 Ainsworth Drive, Suite 109, Prescott, AZ 86301 USA

**Keywords:** Cardiac surgery, Blood management, Point-of-care testing, Quantra, Transfusion

## Abstract

**Background:**

The Quantra QPlus System is a cartridge-based device with a unique ultrasound technology that can measure the viscoelastic properties of whole blood during coagulation. These viscoelastic properties correlate directly with hemostatic function. The primary objective of this study was to assess blood product utilization in cardiac surgery patients before and after the implementation of the Quantra QPlus System.

**Methods:**

Yavapai Regional Medical Center implemented the Quantra QPlus System to aid in their efforts to reduce the transfusion of allogenic blood products and improve outcomes in patients undergoing cardiac surgery. A total of 64 patients were enrolled prior to the utilization of the Quantra (pre-Quantra cohort), and 64 patients were enrolled after (post-Quantra cohort). The pre-Quantra cohort had been managed via standard laboratory assays along with physician discretion for transfusion decisions. The utilization of blood products and frequency of transfusions were compared and analyzed between the two cohorts. (using the Student’s t-test)

**Results:**

The implementation of the Quantra resulted in a change in the pattern of blood product utilization leading to a demonstrated decrease in the amount of blood products transfused and the associated costs. The amount of FFP transfused was significantly decreased by 97% (P = 0.0004), whereas cryoprecipitate decreased by 67% (P = 0.3134), platelets decreased by 26% (P = 0.4879), and packed red blood cells decreased by 10% (P = 0.8027) however these trends did not reach statistical significance. The acquisition cost of blood products decreased by 41% for total savings of roughly $40,682.

**Conclusions:**

Use of the Quantra QPlus System has the potential to improve patient blood management and decrease costs.

**Study registered at ClinicalTrials.gov:**

NCT05501730

## Introduction

Cardiac surgery is associated with perioperative blood loss and a high risk of allogeneic blood product transfusion [1]. Adverse clinical outcomes are associated with high blood product transfusion requirements and reoperation for bleeding [2]. Although various allogeneic blood components and pharmacological agents are available to treat coagulopathic bleeding perioperatively, accurate and timely tests to determine their indications and dosing are needed. This is crucial to avoid unnecessary transfusions, reduce blood product waste, decrease health care costs, and improve patient outcomes.

Patient blood management contributes to the maintenance of hemostasis, minimizing bleeding and decreasing the need for transfusion. Historically, clinicians have relied on conventional laboratory tests to identify the causes of coagulopathy. Conventional tests require blood samples to be transported to the lab which leads to long turnaround times. Further, these tests cannot detect important coagulation defects such as excessive fibrinolysis, platelet dysfunction, or specific coagulation factor deficiencies [3]. As a result, empiric therapy based on clinical judgment is often employed. This strategy can lead to underuse of blood components in some patients, resulting in excessive blood loss and possibly re-exploration, and to overuse of blood components in others, exposing them to unnecessary risks [4]. Measurement of activated clotting time (ACT) has been implemented at the point-of-care however this testing is limited to monitoring of heparin concentrations.

Clinical guidelines developed by the American Society of Anesthesiologists for patient blood management recommend the use of viscoelastic testing (VET) devices in conjunction with goal-directed treatment algorithms to aid in the management of coagulopathic bleeding [5]. Goal directed transfusion algorithms that incorporate whole blood testing, such as with viscoelastic devices, are recommended to reduce periprocedural bleeding and transfusion in cardiac surgical patients in guidelines set by the Society of Thoracic Surgeons (Class I, Level B-R) [6]. Several prospective randomized studies in cardiac surgery have demonstrated the effectiveness of VET in reducing postoperative hemorrhage and certain blood product transfusions [7, 8]. Two technologies have emerged at the forefront of whole blood VET: thromboelastography (TEG System; Haemonetics Corporation, Braintree, MA) and rotational thromboelastometry (ROTEM; Instrumentation Laboratory, Bedford, MA). Both use an adaptation of the classic methodology first reported by Hartert that involves a pin suspended in a cup containing a blood sample [9]. These tests have often been described as point-of-care however, few have been utilized at the bedside of the cardiac patient due to sample processing and pipetting requirements. Recently, cartridge-based versions of these devices have been introduced to address these limitations (Haemonetics’ TEG 6 S; Werfen, ROTEM sigma) [10].

The Quantra QPlus System (HemoSonics, LLC, Durham, NC) is a cartridge-based VET system that uses a novel ultrasound technology [11, 12]. The system has been cleared by the FDA for use in cardiac surgery and its performance relative to other VET platforms has been established [13–17]. The primary objective of this study was to assess the clinical outcomes in cardiac surgery patients undergoing cardiopulmonary bypass before and after the implementation of the Quantra QPlus System at Yavapai Regional Medical Center (YRMC). We hypothesized that the post-Quantra cohort would utilize fewer blood products during surgery and throughout the hospital stay and this would lead to lower costs associated with blood transfusions.

## Methods

### Study Design

**Fig. 1:**
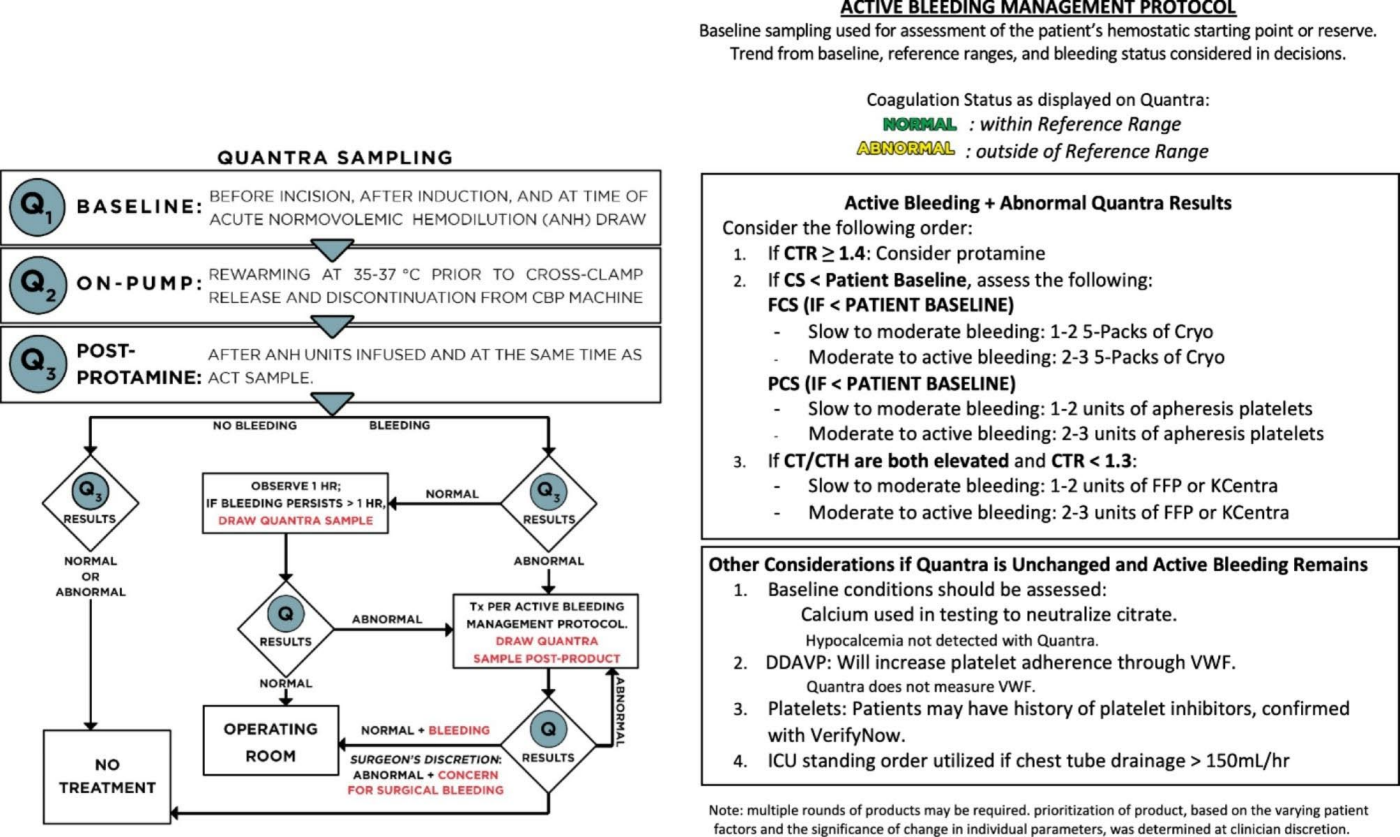
Quantra Treatment Guide Yavapai Regional Medical Center’s Quantra treatment guide utilized in cardiac surgical cases in the post-Quantra group. ACT: activated clotting time; CS: clot stiffness; CT: clot time; CTH: clot time with heparinase; CTR: Clot Time Ratio; DDAVP: desmopressin; FCS: fibrinogen contribution to clot stiffness; ICU: intensive care unit; ml: milliliter; PCS: platelet contribution to clot stiffness; Q_1_: baseline sample drawn in the operating room; Q_2_: sample taken while patient on cardiopulmonary bypass and body temperature at 35–37 degrees prior to cross clamp release; Q_3_: sample taken post protamine administration and after acute normovolemic hemodilution; Q4: Sampling done postoperatively; VET: viscoelastic testing

This study was conducted with the approval of WCG IRB (Protocol 1,287,400). The data was collected from January 2019 through April 2021. All consecutive patients undergoing cardiac surgical procedures at YRMC during a 9-month period from January 2019 to September 2019 were included in the pre-Quantra cohort (n = 64). During that 9-month time-period, utilization of blood products was guided by the institution’s standard of care. Beginning in October 2019, a Quantra-guided transfusion algorithm was instituted at YRMC for the management of patients undergoing cardiac surgical procedures. To allow sufficient time to implement the algorithm and to adjust Quantra trigger values, the post-Quantra cohort period began in May 2020 and concluded in April 2021. During this period, 65 patients underwent a cardiac surgical procedure in which the utilization of blood products was guided by the Quantra QPlus System using an institutional-specific treatment guide (Fig. 1).

During both time periods, there were no major changes/innovations in the surgical procedures themselves that would affect the use of blood products or the decision to return a patient to the operating room. A single surgeon and surgical team at YRMC were responsible for the care of all surgical patients.

For each enrolled patient, the following baseline characteristics were recorded in the study database: gender; age; height/weight/BMI; preoperative medications relevant to coagulation status taken within 5 days of surgery start; preoperative comorbidities; and preoperative diagnosis. Surgery details including surgery type, duration of surgery, time on bypass, and cross-clamp time were also recorded. The following laboratory values were collected preoperatively, during surgery, and postoperatively in the intensive care unit (ICU): platelet count, hemoglobin (Hb) and hematocrit, prothrombin time/international normalized ratio (INR), and partial thromboplastin time. The Quantra QPlus System was used at the point-of-care to evaluate the viscoelastic properties of whole blood by means of the following functional parameters: Clot Time (CT), Clot Time with Heparinase (CTH), Clot Stiffness (CS), Fibrinogen Contribution to Clot Stiffness (FCS), Platelet Contribution to Clot Stiffness (PCS), and Clot Time Ratio (CTR). Quantra parameter results were collected at the following time points: baseline (before incision after induction of anesthesia), on pump (during rewarming), post protamine and after acute normovolemic hemodilution, and postoperatively in the ICU if active bleeding was observed. Decisions to administer blood products were guided by the Quantra Treatment Guide (Fig. 1). Blood products given during surgery and throughout hospital stay were recorded as units received of packed red blood cells (pRBC), fresh frozen plasma (FFP), platelets and cryoprecipitate. For cryoprecipitate, one “unit” or bag consisted of a 5-pack.

### Statistical methods

Statistical analysis was performed using SAS version 9.4. P-values less than 0.05 were considered statistically significant. Categorical variables were summarized as frequencies with percentages and continuous variables as means with standard deviations (SD) or medians with interquartile ranges as appropriate.

The following parameters evaluated in the pre-Quantra and post-Quantra cohorts were compared using the student t-test or z-test: baseline patient characteristics, surgery information, number of units of blood products administered and cumulative costs of transfused blood products. Additionally, the risk analysis as well as the odds ratio were calculated relating the probability of a patient needing more than one blood product postoperatively given the Quantra was not used.

A single patient was identified as an outlier by the Grubb’s test using maximum normalized residuals. This patient in the post-Quantra cohort underwent surgical treatment of an acute myocardial infarction in which the number of blood products utilized in the perioperative and post-operative periods far exceeded that of any other patients in both cohorts. This patient was removed from the study and replaced with the next consecutive patient undergoing cardiac surgery in the post-Quantra cohort.

## Results

### Patient characteristics and surgery information

A total of 128 patients were included in this study with 64 patients included in the pre-Quantra cohort and 64 patients included in the post-Quantra cohort. There was no significant difference between the baseline demographics, comorbidities, laboratory values, or preoperative anticoagulant use in the 5 days preceding surgery in the pre- and post-Quantra cohorts (Table 1). Additionally, there was no significant difference in surgery type, mean surgery duration, number of deaths at discharge, or reoperation rates for bleeding between the pre- and post-Quantra cohorts (Table 2).

**Table 1:** Baseline Characteristics of the Two Cohorts

	Pre-Quantra		Post-Quantra		P-Value
N	64		64		
Women (n, %)	19	29.69%	19	29.23%	0.9522
Age (mean, SD)	71.59	8.46	70.55	9.13	0.4279
Hypertension (n, %)	35	54.69%	49	76.56%	0.0155*
Diabetes (n, %)	13	20.31%	17	26.56%	0.5313
COPD (n, %)	6	9.38%	5	7.81%	> 0.999
Kidney disease (n, %)	2	3.13%	9	14.06%	0.0585
Dyslipidemia (n, %)	20	31.25%	13	20.31%	0.2254
Anemia (n, %)	1	1.56%	3	4.69%	0.6115
Platelets (x1000/uL) (mean, SD)	197.53	53.70	210.20	51.53	0.5602
INR (mean, SD)	1.12	0.11	1.09	0.17	0.2661
aPTT (s) (mean, SD)	32.35	7.22	35.64	16.00	0.1476
Hemoglobin (g/L)	13.94	2.29	14.15	1.88	0.5904
Hematocrit (%) (mean, SD)	42.31	6.40	42.61	5.56	0.7820
Prehospital Medications^1^					
Aspirin (n, %)	12	18.75%	16	24.62%	0.4179
Clopidogrel (n, %)	1	1.56%	1	1.56%	0.9920
Phytonadione (n, %)	1	1.56%	0	0.0%	0.3125
LMWH (n, %)	11	17.19%	16	24.62%	0.2983

^1]^ Prehospital medication is defined as a medication received within the 5 days leading up to surgery

*Statistically significant for two-tailed hypothesis using alpha 0.05

dL: deciliter; g: grams; INR: international normalized ratio; LMWH: low molecular weight heparin; N: total number of patients in cohort; n: total number of patients that received prehospital medication; SD: standard deviation; uL: microliters

**Table 2:** Surgery Types and Duration

	Pre-Quantra		Post-Quantra		P-Value
CABG (n, %)	30	48.88%	37	56.92%	0.2543
CABG + Aortic valve replacement (n, %)	4	6.25%	7	10.77%	0.3576
CABG + Mitral valve replacement (n, %)	4	6.25%	2	3.08%	0.3898
Aortic valve replacement (only) (n, %)	10	15.63%	12	18.46%	0.6672
Mitral valve replacement (only) (n, %)	10	15.63%	5	7.70%	0.1585
Other (n, %)	6	9.38%	1	1.54%	0.0523
**Surgery Duration by Group (min)**					
All surgeries (mean, SD)	207.81	53.39	200.69	48.15	0.5940
CABG (mean, SD)	195.47	30.54	199.84	35.23	0.5940
CABG + Aortic valve replacement (mean, SD)	231.00	8.04	271.86	46.83	0.1249
CABG + Mitral valve replacement (mean, SD)	269.00	67.74	298.00	22.63	0.6051
Aortic valve replacement (mean, SD)	190.20	47.57	157.92	24.04	0.0518
Mitral valve replacement (mean, SD)	182.20	44.13	174.00	18.23	0.7007
Other (mean, SD)	285.33	81.57	186.00	0	0.1534
All surgeries perfusion time (min) (mean, SD)	103.19	30.94	96.58	31.89	0.3140
Reoperation for bleeding (n, %)	2	3.13	3	4.69	> 0.999
Deaths (n, %)	4	6.25	4	6.25	1.00
Total hospital stay (days) (median, range)	8.28	4.2, 30.0	7.31	4.1, 28.5	0.8874

Total hospital stay includes time patients were in the ICU plus progressive care

Deaths refer to mortality status at discharge

*Statistically significant p < 0.05

Other procedures consisted of double valve and ascending aortic repair

CABG: coronary artery bypass grafting; min: minute; n: total number of patients in surgery category; SD: standard deviation

### Blood product utilization

**Fig. 2:**
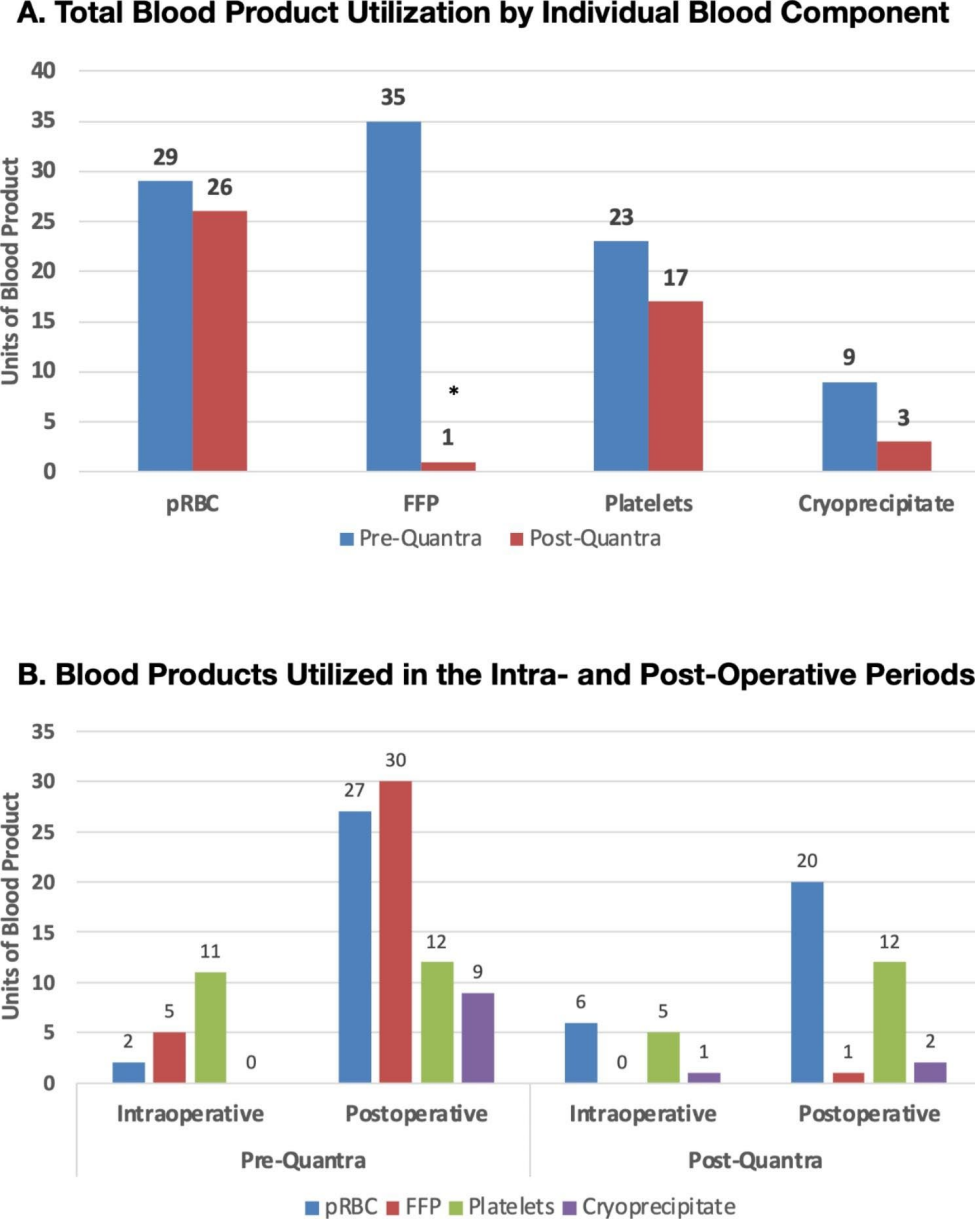
Blood Product Utilization **A**. Total units of blood products utilized during the hospital stay for the two study cohorts. **B.** Utilization of blood products in the intra- and post-operative time periods *Statistically significant p < 0.05 FFP: Fresh frozen plasma; pRBC: Packed red blood cells

Blood product utilization was investigated as both total units of blood products administered across all patients and number of patients receiving blood products during the perioperative and postoperative periods. The total usage of blood products of all types was greater in the pre-Quantra cohort (Pre) compared to the post-Quantra cohort (Post) (Fig. 2A; Table 3 A). This was statistically significant for fresh frozen plasma (FFP) in which 35 units were transfused in the pre-Quantra cohort versus 1 unit in the post-Quantra cohort. Similarly, 9 units of cryoprecipitate were transfused pre-Quantra compared to 3 units in the post-Quantra cohort. By examining the total number of patients in each cohort that received any type of blood product, it is of note, that 29.7% of the post-Quantra cohort required blood products compared to 34.8% of the pre-Quantra cohort (Table 3B). The number of patients receiving at least one unit of pRBC, platelets or cryoprecipitate was similar across both cohorts however significantly more patients in the pre-Quantra cohort received FFP compared to the post-Quantra cohort (14 vs. 1).

**Table 3:** Blood Product Utilization

	Pre-Quantra	Post-Quantra	P Value
**A. Blood products administered (mean units, range)**			
pRBC	0.453 (0, 5)	0.406 (0, 6)	0.8027
FFP	0.547 (0, 6)	0.016 (0, 1)	0.0004*
Platelets	0.359 (0, 4)	0.266 (0, 2)	0.4879
Cryoprecipitate	0.141 (0, 5)	0.0467 (0, 1)	0.3134
Total, all products	1.50 (0, 19)	0.734 (0, 7)	0.0933
B. **Patients receiving blood products (N, %)**			
pRBC	13 (20.3)	13 (20.3)	1.00
FFP	14 (21.9)	1 (1.6)	0.0004*
Platelets	10 (15.6)	12 (18.8)	0.6394
Cryoprecipitate	3 (4.7)	3 (4.7)	1.00
Total, any product	22 (34.4)	19 (29.7)	0.5689

A: Comparison of total number of units of blood products (all types) utilized across all patients intraoperatively and postoperatively. B: Number of patients receiving at least one unit of blood products, *Statistically significant p < 0.05

FFP: Fresh frozen plasma; pRBC: Packed red blood cells

Fig. 2B depicts the relative utilization of blood products in the intra- and post-operative time frames. In both study cohorts, the majority of blood products were administered in the post-operative period.

### Odds ratio analysis

The odds ratio (OR) of a patient receiving a given blood product was also analyzed for each study cohort. The OR a patient would receive fresh frozen plasma was significantly increased in the pre-Quantra cohort (Table 4). Equally, the OR a patient would receive more than one unit of fresh frozen plasma was significantly increased in the pre-Quantra cohort (Table 4).

**Table 4:** Odds of Receiving Blood Products in the Pre-Quantra and Post-Quantra Cohorts

	pRBC	FFP	Platelets	Cryoprecipitate
	OR (CI)	OR (CI)	OR (CI)	OR (CI)
Pre-Quantra	1)1.00 (0.42, 2.37) 2)1.58 (0.53, 4.74)	1)17.64 (2.24, 138.75)* 2) 37.04 (2.16, 636.0)*	1)0.80 (0.32, 2.02) 2)1.93 (0.61, 6.12)	1)1.00 (0.19, 5.15) 2)7.34 (0.37, 145.1)
Post-Quantra	1)1.00 (0.42, 2.37) 2)0.63 (0.21, 1.89)	1)0.06 (0.01, 0.45) * 2)0.03 (0.02, 0.46)*	1)1.25 (0.50, 3.131) 2)0.52 (0.16, 1.64)	1)1.00 (0.19, 5.15) 2)0.14 (0.07, 2.69)

1): Odds patient will receive a given blood product. 2): Odds patient will receive more than one unit of a specified blood product

*Statistically significant p < 0.05

CI: Confidence Interval based on alpha 0.05; FFP: Fresh frozen plasma; OR: Odds ratio; pRBC: Packed red blood cells

### Blood product cost analysis

To compare the costs associated with blood product transfusions between the two cohorts, the following activity-based costs were utilized which incorporate the direct acquisition cost of the product as well as associated indirect costs [18]: pRBC $1112/unit; FFP $578/unit, platelets $1486/unit, and cryoprecipitate $1463/unit. The total cost of each blood product (pRBC, FFP, platelets and cryoprecipitate) was calculated for each cohort along with the total cost of blood products utilized in each cohort. The overall cost of all blood product transfusions combined was higher in the pre-Quantra cohort than the post-Quantra cohort ($99,823 vs. $59,141) (Table 5).

**Table 5:** Cost Comparison of Total Blood Products Utilized

	Pre-Quantra	Post-Quantra	Difference (Pre - Post)
pRBC (total)	$32,248	$28,912	**$3,336**
FFP (total)	$20,230	$578	**$19,652**
Platelets (total)	$34,178	$25,262	**$10,402**
Cryoprecipitate (total)	$13,167	$4,389	**$8,778**
Total Cost	$99,823	$59,141	**$40,682**

Tab 5 the total cost of each blood product was calculated for each cohort along with the total cost of all blood products utilized in each cohort

FFP: Fresh frozen plasma; pRBC: Packed red blood cells

## Discussion

The use of viscoelastic testing devices as part of a goal-directed treatment approach to manage perioperative bleeding has been recommended by several international organizations and clinical guidelines, including the Society of Cardiovascular Anesthesiologists (SCA), the American Society of Anesthesiologists (ASA), the STS/SCA/AmSECT/SABM Update to the Clinical Practice Guidelines on Patient Blood Management, and the European EACTS/EACTA guidelines for cardiac surgery, among others [1, 5, 6, 19]. The aim of this study was to determine the impact of the implementation of the Quantra system on blood transfusions in patients undergoing a cardiac surgery requiring cardiopulmonary bypass at an institution that previously did not use viscoelastic testing. This was a retrospective analysis of how blood product utilization and cost changed after Quantra was introduced. This is one of the first studies demonstrating the clinical performance and utility of the Quantra QPlus System in the cardiac patient population.

Prior to the introduction of the Quantra, the institution’s standard of care for guiding the utilization of blood products relied on results from a panel of routine coagulation tests performed in the hospital laboratory which included aPTT, PT/INR, platelet count, and fibrinogen level. This testing was routinely performed during the last preoperative visit and repeated during the intraoperative phase if coagulopathic bleeding was suspected.

Across pre- and post-Quantra cohorts, patient baseline characteristics and procedural details were similar with approximately half of each cohort undergoing a CABG procedure. Additionally, there was no significant difference in preoperative medications between the pre- and post-Quantra cohorts, defined as patients required to remain on anticoagulant medications within 5 days of surgery.

For all types of blood products, the total number of units administered in the post-Quantra cohort was less than in the pre-Quantra cohort, however, the decrease reached statistical significance for FFP in which 34 units were given in the pre-Quantra cohort whereas only 1 unit was transfused in the post-Quantra cohort. The total reduction in blood product utilization was reflected in lower transfusion costs, with reported saving of over $40,000, representing a 41% reduction in costs vs. the pre-Quantra time period. In contrast, the number of patients receiving any amount of pRBC, platelets or cryoprecipitate was similar across both cohorts suggesting that the number of units of product each patient received tended to be less in the post-Quantra cohort. For FFP, 14 patients received product pre-Quantra and only 1 patient post-Quantra. These findings demonstrate a change in the pattern of blood product administration away from FFP, which is consisted with similar studies on the impact of viscoelastic testing devices in transfusion practices [20, 21]. The use of FFP is associated with several risks including allergic reactions, transfusion-related acute lung injury and transfusion-related circulatory overload [1].

Overall, these results indicate that the use of the Quantra has improved our institution’s transfusion practice by providing a targeted and optimized approach to blood product utilization. The system quantifies and trends (based on serial measurements) the function of the enzymatic clotting factors, presence of residual heparin, as well as the relative function of platelets and fibrinogen, which allows for a streamlined goal-directed treatment algorithm. Additionally, since the system was operated at the point of care and no sample manipulations are required, results were available in less than 15 min, which allowed to rapidly restore hemostatic balance in these patients. The results presented in Fig. 2 further suggest that improved assessment and treatment of coagulopathies in the intra-operative time-period led to reduced blood product transfusions in the post-operative setting.

There were several limitations to this study. The study was conducted at a single institution with a single surgical team involved in the care of patients during the interoperative and immediate postoperative periods therefore, the sample size is limited and results may not account for potential differences in clinical and/or transfusion practices across the diversity of teams and institutions that exist. The post-Quantra time-period was 3 months longer than the pre-Quantra time-period reflecting the additional time it took to attain a similar number of surgical cases due to a reduction in monthly surgical volumes brought on by the COVID-19 pandemic.

The majority of cardiac surgeries performed by this surgical team at this institution were elective, which enabled implementation of the standard recommended time for discontinuing antiplatelet medications prior to surgery for most cases. Results may not be generalizable to an institution with a large number of emergent cases and will require future studies to explore this. This study is one of the first descriptions of improved transfusion outcomes utilizing the Quantra in a facility that did not previously use viscoelastic testing. Further, this study was not intended to provide a comprehensive analysis of the economic impact of implementing Quantra at the point-of-care in a cardiac surgery program. Future studies should be conducted to evaluate the benefits or advantages of the Quantra in comparison with other viscoelastic testing platforms.

## Conclusion

After the introduction of Quantra in the operating room as a component of an active bleeding management protocol for cardiac surgery patients, a reduction in blood product transfusions was observed. Most strikingly was the reduction in utilization of fresh frozen plasma for this cohort of patients. The reduction in transfusions resulted in a cost savings from the required blood products, but also an improvement in patient blood management and care.

## Data Availability

All data generated or analyzed during this study are included in this published article or are available from the corresponding author on reasonable request.

## Abbreviations

CT: Clot time
CTH: Clot Time with Heparinase
CS: Clot Stiffness
FCS: Fibrinogen Contribution to Clot Stiffness
FFP: Fresh frozen plasma
PCS: Platelet Contribution to Clot Stiffness
pRBC: Packed red blood cells
VET: Viscoelastic testing
YRMC: Yavapai Regional Medical Center
